# The Role of Medical Therapies in the Management of Cervical Intraepithelial Neoplasia: A Narrative Review

**DOI:** 10.3390/medicina61020326

**Published:** 2025-02-13

**Authors:** Ioana Cristina Rotar, Dan Boitor Borza, Adelina Staicu, Iulian Gabriel Goidescu, Georgiana Irina Nemeti, Popa Iulia, Melinda Ildiko Mitranovici, Mureșan Daniel, Petca Aida

**Affiliations:** 1 Obstetrics and Gynecology I, Mother and Child Department, “Iuliu Hatieganu” University of Medicine and Pharmacy, 400012 Cluj-Napoca, Romania; cristina.rotar@umfcluj.ro (I.C.R.); dan.boitor@elearn.umfcluj.ro (D.B.B.); goidescu.iulian@elearn.umfcluj.ro (I.G.G.); georgiana.nemeti@elearn.umfcluj.ro (G.I.N.); coropetchi.iulia.elena@elearn.umfcluj.ro (P.I.); daniel.muresan@umfcluj.ro (M.D.); 2 Department of Obstetrics and Gynecology, Emergency County Hospital Hunedoara, 14 Victoriei Street, 331057 Hunedoara, Romania; 3 Department of Obstetrics and Gynecology, “Carol Davila” University of Medicine and Pharmacy, 8 Eroii Sanitari Blvd., 050474 Bucharest, Romania; aida.petca@umfcd.ro; 4 Department of Obstetrics and Gynecology, Elias University Emergency Hospital, 17 Mărăști Blvd., 050474 Bucharest, Romania

**Keywords:** human papilloma virus, cervical intraepithelial neoplasia, personalized treatment

## Abstract

Cervical cancer and its precursors (cervical intraepithelial neoplasia (CIN)) represent a current major public health concern. Currently, the treatment of choice for patients with HSILs (high-grade intraepithelial lesions) is surgical treatment—LEEP or cold-knife conization—except for in pregnant women, where it may have significant future consequences. In this paper, we aim to review the current evidence regarding the efficacy of non-surgical approaches for CINs. Therefore, we searched Google Scholar and PubMed for papers on CIN treatments; 91 studies published in English were included in the analysis. The results of the reviewed studies were variable depending on the agent and methodology used. Overall, the remission rates of CIN II ranged from 43 to 93%. However, for some agents, the results were contradictory. Once topical agents have been proven to be effective, they could be used as an alternative to surgical methods in treating HPV-associated CIN, with fewer adverse effects. The use of local agents could allow for more personalized treatments for patients with CINs. Future directions were also sought.

## 1. Introduction

Cervical intraepithelial neoplasia (CIN) refers to squamous epithelial lesions of the inferior genital tract that are precursors to cervical cancer [1]. Globally, invasive cervix cancer is the second most common cancer among women [2,3]. The American College of Obstetricians and Gynecologists (ACOG) emphasizes the importance of the management of abnormal screening results using the current American Society for Colposcopy and Cervical Pathology (ASCCP) guidelines [4]. The current guidelines for cervical cancer recommend personalized management based on the risk stratification of the patients [4].

The most important pathogenic factor in the development of CINs is a persistent infection with human papillomaviruses (HPVs) [5,6], which has the highest prevalence in young women, mostly between 25 and 35 years old. The association between HPVs, cervical dysplasia, and cervical cancer is stronger for high-risk HPV types (16 or 18) [5].

Due to cancer risk, CINs are divided into two categories: low-grade intraepithelial lesions (LSILs, formerly called CIN I and p16-negative CIN II) and high-grade intraepithelial lesions (HSILs, formerly called CIN III and p16-positive CIN II). The spontaneous regression rate in cases when CIN I is histologically proven is high, up to 85% depending on the patient’s risk factors such as age, HPV infection or smoking habits [7]. On the other hand, spontaneous regression in CIN III is possible, but less probable than in CIN I. However, the risk of developing invasive cancer in the absence of a surgical excisional procedure substantially increases to 5–22%; moreover, cervical cancer represents 4% of total cancers [7].

The current management for cervical dysplasia involves surgical procedures such as lasers, electrosurgery, cryosurgery or a classic excision of the abnormal or precancerous tissue due to an HPV infection [8]. Taking into consideration the side effects of the surgical therapy that affect the obstetrical future of the patient, such as an increase in the premature rupture of membranes, preterm birth, increases in neonatal intensive care unit admission rates, and perinatal mortality, there is an increased interest in the use of chemical topical agents. Even if the therapies are still in the experimental phase, there are some that have been used successfully in the management of vulvar dysplasia due to an HPV infection [9,10].

However, there has been no significant reduction in mortality or morbidity in the past 10 years, and the therapeutic approaches are still very diverse [11].

In our opinion, these treatments can be used in patients with CIN I and cervical condyloma after the colposcopic exclusion of a high-grade lesion if the lesion is fully visualized and the junction is visible. Several classes of agents have been used, such as antineoplastics, immunomodulators, antiviral drugs, herbal extracts, synthetic chemical preparations, hormonal drugs, or a combination of these agents.

The aim of this review is to identify topical agents for local use in CIN, which could be an alternative approach until the emergence of therapeutic vaccines.

## 2. Materials and Methods

**Figure 1:**
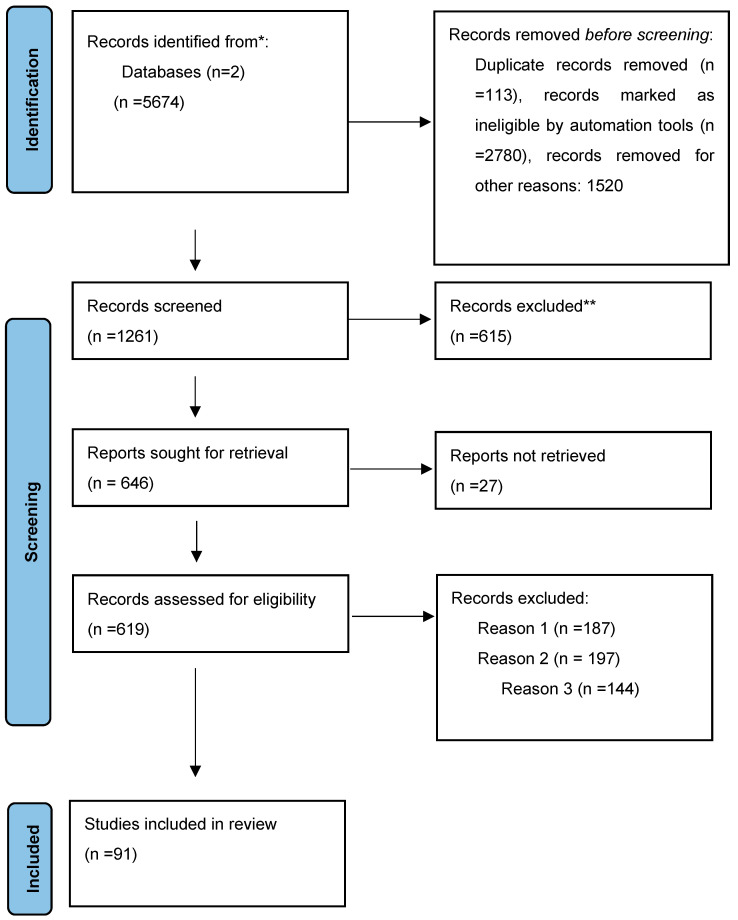
Flow diagram of paper selection. * The number of records identified from Google Scholar and PubMed databases. ** Records excluded by a human. Reason 1—Records excluded publications in a language other than English. Reason 2—Records excluded by a human reviewer due to inaccurate or inappropriate titles. Reason 3—Records excluded based on the study s research design.

For the current review, we searched PubMed and Google Scholar for all available original articles on treatments for CIN (see Figure 1). We chose databases and key words (human papilloma virus; cervical intraepithelial neoplasia; personalized treatment) to exclude relevant titles from 1996 to 2024. As automatic tool we used Abstrackr, and excluded duplicates and irrelevant abstracts. This is a free, semi-automatic, open-source application, which allows for citation screening; the reviewers then only screened articles considered relevant by the software. Screening full text manuscripts, irrelevant trials have been excluded. In the full text screening, we used the snowball method, searching for citations from included manuscripts.

Based on this search, we identified a total of 5674 articles. The inclusion criteria for our search were key words, full text manuscripts, and the presence of a proper study design (clearly presented, with properly explained inclusion and exclusion criteria, dosage and administration of medication explained, follow-up described, results clearly presented, or reviews based on clinical trials and relevant information). After excluding the articles published in languages other than English, or with inaccurate or inappropriate designs, or where only the abstract was available, 91 studies remained and were included in the current review (Figure 1).

## 3. Topical Agents

### 3.1. Topical Chemotherapy

#### Topical 5-Fluorouracil

5-fluorouracil is an anticancer agent that inhibits DNA and therefore slows tumor growth [12,13]. Although it was introduced over 30 years ago, 5-fluorouracil continues to be one of the most widely used anticancer drugs. It is used in the treatment of several different malignancies, alone or in combination with other therapeutic agents [14].

Topical 5-fluorouracil (5-FU) is used for the therapy of skin infection with HPVs, including genital lesions [9,10]. The treatment seems to be associated with severe adverse effects such as local pain. These side effects are likely a dose-related response due to multiple daily applications [14].

Rahangdale et al. (2014) performed a prospective randomized trial in woman aged 18–29 years with CIN II, who were divided into observation and treatment with intravaginal 5-FU groups. Regression of CIN II was observed in 93% of the women in the 5-FU group—who were given a topical 5-FU dose every 2 weeks—and 56% of the women in the observation group. At the 6-month follow-up, the women in the 5-FU group were twice as likely to be HPV negative compared to the women in the control group [15]. 5-FU is an off-label medication used in vaginal intraepithelial neoplasia treatment and has been shown to play an important role in the recurrence of CINs [12,16].

In addition, Fiascone et al. (2017) demonstrated that intravaginal 5-fluorouracil could be used to treat VAIN (vaginal intraepithelial neoplasia) with a high success rate [17].

### 3.2. Immunotherapy

A new targeted therapy is represented by antibody–drug conjugates (ADCs), which are a modality that can kill tumor cells in a more effective way. Immunotherapy consists of a monoclonal antibody that targets a tumor-specific antigen not generally found on normal cells. This can be a promising treatment for use in cervical cancer [10,18,19,20].

#### 3.2.1. Imiquimod (Aldara^®^)

Imiquimod is an imidazoquinoline-fused drug [5,6] that has been demonstrated to have potent immunomodulating, antiviral, and antiproliferative activities. Clinical studies have shown that imiquimod can induce a significant reduction in viral loads [21,22].

Thus, the enhancement of the immune response mediated by imiquimod may have potential applicability in the treatment of cervical dysplasia, as a persistent HPV infection is strongly linked to the development of CIN and malignancies. Imiquimod has shown significant efficacy in treating vulvar intraepithelial neoplasia (VIN) and vaginal intraepithelial neoplasia (VAIN) [23].

In Grimm et al.’s study, fifty-nine patients with untreated CIN II/CIN III were randomly allocated to a 16-week treatment with vaginal topical imiquimod or placebo. The vaginal applications of 6.25 mg imiquimod were administered as follows: one vaginal suppository weekly for the first 2 weeks, two vaginal suppositories weekly for the next 2 weeks (week 3 and 4), and then three vaginal suppositories weekly until the end of the 16-week treatment period. The randomized controlled trial performed by Grimm demonstrated local tolerance without significant adverse effects with statistically significant regression (73%) in the study group compared with the placebo group (39%) (*p* = 0.009). Complete histologic remission was observed in 47% of the patients receiving the imiquimod, which is higher than in the placebo group (14%) (*p* = 0.008) [24].

Cutaneous side effects were observed, such as vitiligo, psoriasis, or pemphigus, but in rare situations, and these reactions were reversible [25]

Toll-like receptor 7 and 8 (TLR 7 and 8) and nuclear factor-kappa B (NF-kappa B) were found to play an important role. The major biological effects of imiquimod are mediated through TLR and NF-kappa B activity [26].

Pachman’s study included 56 patients and compared standard excisional treatment vs local applications of imiquimod. No differences were observed in CIN recurrence after 2 years between the 2 groups. Side effects after imiquimod were significantly higher than after excision, such as fatigue, fever, vaginal discharge, and headache. The study of Pachman et al. had some limitations [27]. First, it had a small sample size—their goal was to recruit 152 patients, but they had only managed to enroll 56 patients. A possible explanation was the frequent visit routine, demanding five vaginal applications of imiquimod followed by two clinic visits daily [27].

#### 3.2.2. Interferons

Interferons (IFN) are low-molecular-mass glycoproteins released by host cells that belong to cytokines, which are molecules used for crass-talk between cells in order to enhance the protective activity of the immune system to help eradicate pathogens [28]. They also have antiproliferative effects, and are used in the treatment of cancer, as well as being protective effects against radiation. The application of local IFNs could be useful in the initial phase of a viral infection to stop viral replication [29]. Not many clinical trials have been carried out on interferons’ relevance in CIN [29].

Yua Go’s study in 2024, carried out on 112 patients, compared argon plasma coagulation with interferon. The APC group included 77 patients, 35 patients being treated with interferon. A significantly higher HPV clearance after a 12-month follow-up was observed in the APC group—significantly higher compared to in the interferon group (87.67% vs. 51.52%, *p* < 0.05). Treatment with APC is more effective compared to interferon, with a significantly higher cure rate in the APC group (79.22% vs. 40.0%) [30].

#### 3.2.3. Granulocyte–Macrophage Colony-Stimulating Factors (GM-CSFs)

GM-CSF is a hematopoietic cytokine that helps repairing and making more white blood cells. Antigen-presenting cells (APC) in CIN were evaluated in two clinical trials with 15 patients with LSIL and 11 healthy women. GM-CSF local applications were performed; all patients with CIN presented an elevated immune response after the application, NK and T cells produced interferon gamma and APC was increased. No toxic effect was recorded during the follow-up (until 30 months). Immune response against HPV16 after GM-CSF applications was recorded in all women with CIN. Validation is needed due to the limited number of subjects [31].

These cytokines are produced by keratinocytes. The levels of GM-CSFs are correlated with tumor-associated DCs. Moreover, in vitro, DCs have been shown to specifically induce the apoptosis of keratinocytes transformed by HPVs, whereas normal keratinocytes are not affected [32].

### 3.3. Anti-Viral Medications

#### 3.3.1. Cidofovir

Cidofovir, an acyclic phosphonate nucleoside active against DNA viruses, is recognized as an effective treatment for genital and extra-genital HPV lesions [33]. Cidofovir in topical applications has been shown to have various indications: the treatment of herpes lesions in immunocompromised patients that are resistant to acyclovir, Molluscum contagiosum, adenovirus infections, and cutaneous and genital warts, which are not associated with HPVs [34].

Cidofovir 1% gel can inhibit cervical dysplasia lesions. In a study by Van Pachterbeke et al., cidofovir was observed to be active against CIN II lesions in women. In his study, histologically complete remission was demonstrated in 61% of the cidofovir group compared with 20% in the placebo group. The failure to completely regress could be caused by the insufficient penetration of the drug into all targeted cells, or the severity of the lesion. The short observation time of 6 weeks is another important weakness. Most of the studies that used a longer study period (3 to 6 months) found more complete responses [35].

Thus, it must to be verified whether a prolonged exposure to topical drugs could provide better outcomes. The Van Pachterbeke et al. study grouped the results of the CIN II and CIN III patients together, so a better response could be expected in an exclusively CIN II population treated with imiquimod and cidofovir, based on the higher remission rates for CIN II patients in the studies with separate CIN II and CIN III datasets [ 35].

#### 3.3.2. Imunovir (Inosine Pranobex)

A relatively safe immunomodulator, inosine pranobex (Imunovir), was introduced as a potential treatment method for HPV infection and cervical cancer, which can be tailored to each patient’s condition [36].

More recently, inosine pranobex has been developed as an oral therapy, and this has increased success rates in the treatment of HPV lesions with classical therapy [37].

Tay’s study showed that inosine pranobex was superior to the placebo in improving the vulval epithelial dysplasia caused by subclinical HPV infections. The participants in this study were women with chronic pruritus vulvae. This study revealed that inosine pranobex induced significant regression in the dysplasia in almost two-thirds of the treated patients. The compliance with administering the oral medication and the low adverse drug reactions make this an appropriate treatment for women with a symptomatic vulval HPV infection [38].

There are no studies in the literature demonstrating that inosine pranobex can eliminate HPVs from cervical lesions.

### 3.4. Vitamin A Compounds

#### Retinoids

Retinoids are synthetic derivatives of vitamin A. First- and second-generation retinoids have the flexibility to bind with several retinoid receptors, compared to third-generation retinoids [39].

An important role of retinoids is their involvement in epithelial cell proliferation and differentiation (which are essential for cell growth, differentiation, and cell death). Retinoids have been studied as a prevention option for various cancers [39]. Retinoids may have a role in inducing apoptosis and differentiation [40].

Alvarez et al. reported the results of a clinical trial of aliretinoin (9-cis-retinoid acid) in 114 women with CIN II or III. The participants were randomized into three groups that received daily oral doses of aliretinoin (50 mg or 25 mg) or a placebo for 12 weeks. After medical treatment, the histological response was zero. There was no significant difference in the regression rate between the placebo (32%), 25 mg (32%) and 50 mg aliretinoin (36%) groups. Headache was the most frequent adverse effect [41].

Ruffin et al. performed a randomized study in which 175 women with CIN II or III were given daily doses of all-trans retinoic acid (atRA), via vaginal application, at doses of 0.16%, 0.28%, or 0.36%, or a placebo for 4 consecutive days. A biopsy was performed 12 weeks after entry into the trial to evaluate the outcome. No significant difference was observed in response rate among the study groups. However, the women with CIN II in the treatment group showed a higher incidence of disease regression compared to CIN III [42]. No sign of therapeutic efficacy was obtained when using retinoids for preventing the progression of CINs, according to a recent meta-analyses [43].

### 3.5. Trichloroacetic Acid

Trichloroacetic acid is an analogue of acetic used for its destructive effect on the skin and mucosa, causing burns and physical destruction through protein coagulation.

Gleiser et al. provided encouraging data on the local use of trichloroacetic acid for the treatment of LSILs and HSILs, with the regression of the lesions observed at 6 weeks post-treatment [44]. A histologically complete remission was observed in a phase II clinical trial conducted by Schwameis et al. (2022; phase II clinical trial on Topical Trichloroacetic Acid used in CIN), with the clearance of high-risk HPV infection. Mild discomfort was reported in 36 cases of the 102 patients included, with no severe adverse events [45].

### 3.6. Photodynamic Therapy

#### 5-Aminolevulinic Acid

Photodynamic therapy (PDT) is a relatively new treatment modality, used in epithelium neoplasias. PDT is based on photoreactive drugs that preferentially concentrate in tumors [46,47].

Clinical studies on the treatment of CINs using PDT are limited, but the topical application of 5-ALA in the PDT of CINs is correlated with disappointing results [48].

### 3.7. Natural Therapies

Natural therapies, including the administration of propolis, turmeric, other plants (e.g., green tea, Sanguinaria canadensis, glycyrrhizin acid (sweet wood extract), and aloe vera), or B vitamins, do not have enough evidence behind them to be recommended for the treatment of CINs [49]. Podofilox 0.5% is a mitotic agent developed from podophyllin resin. Unlike podophyllin, it is more stable, and causes fewer systemic effects [50].

A local preparation based on a Coriolus versicolor extract (Papilocare^®^, Gedeon Richter, Targu Mures, Romania) has also recently appeared in Romania. The studies on this preparation are in the beginning stages. Preliminary data were presented in 2019 from groups of 84, 66 and 29 patients corresponding to high-risk HPV 16-18-31 diagnosed with ASC-US or LSIL, which showed a significant reduction in lesions [51].

#### Polyphenon E

Sinecatechins is another topical drug used for condyloma acuminata that was recently approved and has shown promising results in some clinical trials. Its clinical efficacy is due to the presence of catechins that are known to interfere with multiple cellular pathways, providing modes of action that may result in better treatment outcomes. After concentrating the eluate, more than 90% (by weight) of the resulting powder consisted of catechins [52]. The most abundant and the most important active catechin compound is (epigallocatechin gallate) (EGCG), which accounts for >55% of the total polyphenol content, according to the Veregen prescription information [53].

The first botanical drug to be licensed for topical use to treat external genital and perianal warts in immunocompetent patients was Polyphenon E (Veregen^®^, MediGene AG, Martinsried, Germany). This product is an extract made from the leaves of green tea, Camellia sinensis, and contains tea polyphenols. Catechins account for more than 85% of the tea polyphenols [54,55]. Polyphenon E’s most important component is epigallocatechin gallate, with high biological activity, including antitumor, immunostimulatory, and antiproliferative effects against keratinocytes that are infected with the virus [52]. Moreover, catechins have strong antioxidative properties, which could inhibit the transcription of HPV proteins, as Rösl et al. have demonstrated [52].

The efficacy of using Polyphenon^®^ E (MediGene AG, Martinsried, Germany) in the treatment of genital and perianal warts has been proven in a number of studies [56,57]. Stockfleth et al. showed that more than 50% of the patients treated with Polyphenon^®^ E 15% and 10% ointments exhibited complete clearance of all the baseline and new genital and perianal warts after self-applying the topical treatment three times a day for up to 16 weeks. The same study revealed that the rates of recurrence 12 weeks after the treatment were between 4% and 6% [56]. Tatti et al. demonstrated a similar efficacy for a topical Polyphenol^®^ E treatment, with a rate of complete clearance of the anogenital warts between 53 and 55%, while the recurrence rate was below 7% [58]. Meta-analysis studies comparing different self-applied topical treatments revealed that products containing catechins have better clearance and recurrence rates (46.8–59.4% clearance rate; 4.1–11.8% recurrence rate) compared to imiquimod 5% and podophyllotoxin, and are also well tolerated by patients [55].

Recently, researchers showed an increased interest in the use of sinecatechins in cancer therapies. A case report in 2013 by Gupta et al. detailed the case of a 45-year-old patient diagnosed with vulvar intraepithelial neoplasia, warty subtype, with HPV and CIS; after being treated for 8 weeks with imiquimod 5% ointment without any result, the patient was prescribed Veregen (sinecatechin 15%) ointment three times a day for six weeks. At the end of the 6 weeks, local examination of the patient showed a complete resolution of the lesions, with only mild scarring of the vulva, which was accompanied by negative biopsies for dysplasia or carcinoma [59].

Ahn et al. studied the application of green tea extracts such as, Polyphenon^®^ E, on human cervical lesions. Overall, the topical application of the Polyphenon^®^ E showed clinical efficacy in 74% of patients. A better outcome was observed in combination with the oral administration of Polyphenon^®^ E capsules, with a positive response in 75% of patients [60].

The use of Polyphenon^®^ E 15% and 10% ointments led to recurrence rates of 5–9% and 4–1%, respectively [61]. The other treatment modalities resulted in recurrence rates between 5% and 65%. Cryotherapy showed a high clearance rate, but the risk of recurrence was about 20–40%. Also, imiquimod 5% cream, associated with podofilox, demonstrated good efficacy, but the recurrence rates ranged from 13% to 19% and up to 91%, respectively [62].

The available topical therapies are listed in Table 1.

## 4. Discussion and Future Directions

Cervical cancer is an important public health problem. In 2018, the European Society of Gynecological Oncology (ESGO), European Society of Pathology (ESP), and European Society for Radiotherapy and Oncology (ESTRO) developed evidence-based recommendations for the management of patients with cervical cancer [63]. Due to the frequent occurrence of spontaneous CIN II regression and possible adverse effects of treatment on future pregnancies, conservative management is the proper approach. However, there is a risk of progression to cervical cancer, so personalized approaches are needed [64]. For postmenopausal women, the large loop excision of the lesion remains the most cost-effective treatment [65,66,67].

All the proposed local treatments were evaluated, and discrepancies were found in the results for the topical agents between studies. 5-fluorouracil has shown good results in terms of CIN remission. Moreover, it is known to be an efficient anticancer drug for use in chemotherapy to treat various types of neoplasms. Polyphenon^®^ Eointment is an efficacious and safe topical treatment for genital warts. Its use needs further clinical investigation.

Chemical topical agents may be a therapeutic option, especially in young women where there is a natural clearance of HPVs and where surgical treatment is not indicated. Undoubtedly, after the implementation of national HPV vaccine programs, the incidence of HPV-induced lesions will decrease, but until then, all therapeutic options are valuable.

We summarize the recurrence rates and clinical outcomes according to the studies included, while the main limitation of our review is the lack of long-term follow-up, besides the heterogeneity of the included studies.

In Rahangdale et al.’s work (2014), after the 6-month follow-up, the regression of HPV was significantly higher in the 5-FU group compared to the control group [15]. In Grimm et al.’s study with imiquimod, complete histologic remission was obtained in 47% of the imiquimod group (47%), but long-term follow-up was not performed [24]. In Pachman’s study, after 2 years of follow-up, there were no differences in dysplasia recurrence between the two groups, that is, study and control [27].

Via GM-CSF treatment, Hubert Pascale et al. obtained good immune responses in LSIL and HPV16 after a 30-month follow-up [31].

Van Pachterbeke et al. used Cidofovir, an antiviral medication, with a 61% histological clearance of CIN compared with the control group (20%), but the follow-up period was very short, at 6 weeks [35].

In Alvarez et al.’s study on retinoids, comparable outcomes were observed with the control group in which loop electrosurgical excision was carried out after 12 weeks of follow-up, with a 32% regression rate [41]. The same was obtained in Ruffin et al.’s study [42], but compared with placebo, an extensive meta-analysis showed no evidence of therapeutic activity following the retinoids treatment of CIN [43]

Gleiser et al. showed a better outcome following treatment with trichloroacetic acid with short-term follow-up (6 weeks) [44]. The same good results with clinical complete remission were recorded by Schwameis et al. after 6 months of follow-up [45].

In their work on natural therapeutic topical compounds, after 6 months of follow-up, Serrano et al. observed normal cytology in a significant proportion (88%) [51]. In Stockfleth et al.’s study on Polyphenon^®^ E, the rates of recurrence after 12 weeks of follow-up were between 4% and 6% [56]. The same was observed by Tate et al., at <7% [54]. Recurrence after the use of Polyphenon^®^ E depends on dosage [62].

In Yua Go’s study with interferon, the researchers did not get the expected results with a better clearance rate after argon plasma treatments at a 12-month follow-up [30]. Sikorski et al. (2003) observed that IFN-gamma intracervical injection (used on 20 women with CIN1 or CIN II) seems to be a valuable method, yielding significant treatment-related regressions (*p* < 0.05) compared to spontaneous regression [68]. Michelin (2015) concluded that immunotherapy with IFN-α, applied subcutaneously once weekly in 17 patients with CIN II-III), is a viable clinical treatment [69].

Until now, this approach to topical treatment has seen no clinical application given the lack of proper validation. Our review found heterogeneous results for many treatments. In addition, new directions towards a personalized approach are being taken into account. We only want to underline their value and the need for further evaluation.

As a future direction, in terms of efficacy and safety, delivering a smaller amount of an active substance at the local site is ideal. Conventional dosage forms are associated with leakage, and frequent, repeated administration is necessary. Novel carriers can be engineered with bio-adhesive properties in order to achieve the correct therapeutic level for a prolonged period of time [66,67].

Photodynamic therapy is a pathogenetically substantiated treatment. The reproductive outcomes in treated patients have demonstrated the high effectiveness of chlorin e6-mediated fluorescence-assisted systemic photodynamic therapy. Recurrence occurred in 3.3% to 8.9% during the follow-up period of 2 years. This treatment had high efficacy and a good safety profile: the reported adverse events were mild, with rapid recovery after the therapy. Vaginal discharge and a burning sensation were identified as the most common side common effects. Additionally, the method was evaluated for its effects on fertility, and it was found that its use is safe [70,71]. The effectiveness of PDT may vary depending on the type of PDT agent used, as well as the duration of treatment, the dosage of medication and frequency of the PDT administration, the location and severity of the lesions and the host’s immunological response [72].

Molecular biomarkers are promising prognostic factors that can lead to the correct risk stratification of patients. Various biomarkers such as squamous cell carcinoma antigen (SCC-A), serum YKL-40, circulating HPV DNA and circulating micro-RNAs have shown potential utility as non-invasive biomarkers [4,73]. Viral (HPV DNA and HPV E6/E7 mRNA) and cellular markers (Cyclin-Dependent Kinase Inhibitor 2A (CDKN2A)/p16ink4a, together with the proliferation marker Ki-67, region 3q26) have been evaluated as biomarkers to improve the screening and prognosis of CC [10,18]. The vascular endothelial growth factor (VEGF) serum levels were also shown to be a viable biomarker [74]. Interleukine-6 (IL-6) may be involved in the progression of CIN to cervical cancer, and could offer a treatment biomarker for this disease [75].

Robust molecular markers are needed in order to predict therapy response and survival, and they may help in the development of new targeted therapies [76,77].

In terms of future directions, along with topical compounds, researchers are focusing on targeted therapy, using, for example, chimeric antigen receptor-modified T cells (CAR T), which is a promising approach. This is a kind of immunotherapy that utilizes the ability of the immune system to recognize specific antigens on the CC cell’s surface [11,78]. Anti-programmed death-1/anti-programme death-ligand 1 (Anti-PD-1/PD-L1) antibodies are immune checkpoint inhibitors, which have shown remarkable clinical efficacy in certain gynecological malignancies [11,79,80,81,82]. Knocking down human papilloma virus oncoproteins should also not be overlooked [77].

Another interesting therapeutic option is non-invasive physical plasma treatment (NIPP), which is based on slowing cell growth due to DNA damage, apoptosis, and cell cycle arrest [83].

Antioxidative sodium selenite combined with citric acid and silicon dioxide contained in a medical device has proven effective against histologically proven CIN II as well as p16-positive CIN I [21]. Other therapeutic avenues include angiogenesis inhibitors, together with immune checkpoint inhibitors; immune system enhancement, including agents targeting defective DNA repair, such as oli ADP ribose polymerase (PARP) inhibitors [81,82,83,84]; or live vector-based vaccines, which represent a genome-editing method based on the endogenous repair mechanism [85]. Anti-angiogenic drugs such as Bevacizumab can improve tumor infiltration by immune cells. In addition, cemiplimab, together with local chemotherapy, has shown efficacy in treating CINs [81,82]. Safe gene delivery strategies are also being developed [78,79].

Nanomaterials have emerged as another chemotherapeutic avenue [74,82]. Among them, chitosan nano-capsules containing Chlorocyan-aluminum phthalocyanine as a photoactive agent seem to be feasible and safe [86].

Future research is needed in the areas of artificial intelligence (AI) and machine learning (ML), these being critical components of digital management in cervical cancer care. These technologies offer personalized healthcare services that could revolutionize the field [4]. Large language models (LLMs) show the potential to provide intelligent question-answering with reliable information about medical queries in clear and plain English, which can be understood by both healthcare providers and patients [87].

Alternative medicine therapies are being frequently used to treat cancer in so-called integrative oncology [88]. Fuzheng Jiedu, a traditional Chinese medicine formula, was shown to be effective against persistent HPV infections and to reduce the HPV conversion rate in patients with infertility. Traditional Chinese medicines have been shown to improve symptoms and can increase CD3^+^, CD4^+^, and CD4^+^/CD8^+^ levels. The effect of this combined therapy is stronger than that of TCM or classical medicine alone [67].

New studies on natural compounds, such as Papilocare^®^ vaginal gel, have shown higher regression rates of LSIL cervical dysplasia caused by this treatment than spontaneous regression rates [89]. The same situation was observed in the PALOMA trial, where Papilocare^®^ demonstrated efficacy in treating human papillomavirus (HPV) infection associated with LSIL lesions [90]. The treatment with Coriolus versicolor has raised expectations notably [91].

## 5. Conclusions

Topical therapies can play an important role in secondary prevention by safely treating precancerous lesions, which can be a great advantage compared to surgical methods that are burdened by many complications in the short and long term.

In particular, in young women where the preservation of fertility is the main goal, reproductive outcomes and recurrence frequency must be evaluated after targeted personalized treatments. Various options have been evaluated, but they still require validation. A risk stratification of the patients and a strict follow-up are needed.

## Figures and Tables

**Table 1:** Topical agents used for treating CINs.

Authors	Topical Agent	Study Group	Control Group	Results (Study/Control)	Adverse Effects	Limitations
Rahangdale et al. (2014) [15]	Chemotherapy for CIN 5-fluorouracil	31	29	93%/56% *p* < 0.05	No moderate or severe side effects	
Fiascone et al. (2017) [17]	Chemotherapy for VIN 5-fluorouracil	35	20 (excision), 9 (ablation)	74% rate of success (57%/41%)	16% experienced irritation and dyspareunia	
Grimm et al. (2012) [23]	Immunotherapy Imiquimod	25	24	Regression: 73%/39%, *p* = 0.09 Complete remission: 47%/14%, *p* = 0.08	No high-grade side effects	
Pachman et al. (2012) [27]	Immunotherapy Imiquimod	28	28 (standard treatment)	Recurrence rate: 14%/23%	Chills, fatigue, fever, headache, myalgias, and vaginal discharge	
Yua Gao et al. (2024). [30]	Immunotherapy Interferons	35	argon plasma coagulation 77	significantly higher cure rate in the APC group (79.22% vs. 40.0%	No high-grade side effects	No reliable data about the patients
Hubert s et al. (2004) [31]	Immunotherapy GM-CSFs					Animal model
Van Pachterbeke et al. (2009) [35]	Anti-viral therapy Cidofovir	23	25 (placebo)	Complete remission: 60.8%/20%, *p* < 0.05	Side effects were limited	
Tay et al. (1996) [38]	Anti-viral therapy Imunovir	22	24 (placebo)	63.5%/16.7%, *p* = 0.005	Adverse reactions were mild and self-limiting	
Alvarez et al. (2003) [41]	Anti-viral therapy Imunovir	114 patients enrolled		No significant difference between the groups	Headache	Not clear how many patients were in the study and control groups
Ruffin et al. (2004) [42]	Retinoids	131 patients divided into three groups: low, mild, and high doses of retinoids	44	Neither approach was more effective than placebo	No side effects	
Geisler et al. (2016) [44]	Trichloroacetic acid	179 CIN II-III patients	62 CIN I patients	Remission rate: 80.3%/82.3% High remission rate in both groups	No major side effects	
Cai et al. (2024) [47]	5-aminolevulinic acid-based photodynamic therapy (ALA-PDT)	81 patients, 24-month follow-up	Same 81 patients, 36-month follow-up	Remission rate: 95.83%/100%	No major side effects	No placebo group Difficult to follow the results
Hillemanns et al. (2015) [48]	Hydrochloride-based photodynamic therapy	241, of which 19 received 5% hydrochloride	21	Good remission rate: 95%/57%	Local self-limiting adverse reactions including discharge, discomfort, and spotting	
Serrano et al. (2019) [51]	Natural therapy Papilocare	Atypical squamous cell of undetermined significance and low squamous intraepithelial lesion (LSILs) in high-risk population (66) and populations with HPV (29); total of 95	84 controls with ASCUS or LSILs	Remission with Papilocare: 88%/56%	No major side effects	
Stockfleth et al. (2008) [57]	Natural therapy Polyphenon E	503	Not clear	53%/37%	No major side effects	The control group was not clear
Tatti et al. (2010) [58]	Natural therapy Polyphenon E	401 (10% Polyphenon E)/397 (15% Polyphenon E)	207	53.6% (10% Polyphenon E)/54.9% (15% Polyphenon E)/35.4% (control)	No major side effects	
Ahn et al. (2003) [61]	Natural therapy Green tea extracts (Polyphenon E)	51	39	69%/10%	No major side effects	

## Data Availability

No new data were created or analyzed in this study.
